# Multi-Task Assignment Method of the Cloud Computing Platform Based on Artificial Intelligence

**DOI:** 10.1155/2022/1789490

**Published:** 2022-10-13

**Authors:** Yongchang Zhang, Panpan Geng

**Affiliations:** ^1^Jiangsu Vocational Institute of Architectural Technology, Xuzhou 221116, China; ^2^Xuzhou Intelligent Machine Vision Application Technology Engineering Research Center, Xuzhou 221116, China

## Abstract

To realize load balancing of cloud computing platforms in big data processing, the method of finding the optimal load balancing physical host in the algorithm cycle is adopted at present. This optimal load balancing strategy that overly focuses on the current deployment problem has certain limitations. It will make the system less efficient and the user's waiting time unnecessarily prolonged. This paper proposes a task assignment method for long-term resource load balancing of cloud platforms based on artificial intelligence and big data (TABAI). The maximum posterior probability for each physical host is calculated using Bayesian theory. Euler's formula is used to calculate the similarity between the host with the largest posterior probability and other hosts as a threshold. The hosts are classified according to the threshold to determine the optimal cluster and then form the final set of candidate physical hosts. It improves the resource utilization and external service capability of the cloud platform by combining cluster analysis with Bayes' theorem to achieve global load balancing in the time dimension. The experimental results show that: TABAI has a smaller processing time than the traditional load balancing multi-task assignment method. When the time is >600 s, the standard deviation of TABAI decreases to a greater extent, and it has stronger external service capabilities.

## 1. Introduction

Cloud computing is the research hotspot direction in distributed computing. It inherits the characteristics of grid computing, utility computing, and other classic distributed computing frameworks [1–5]. Cloud computing provides users with on-demand infrastructure services, platform services, and software services through the network [6]. Infrastructure services are the most important capabilities and features of cloud computing. In a cloud resource pool, many hosts are usually cited and resources and services are provided based on virtual machine technology. The available resources of all physical hosts are dynamically adjusted during the process of providing services on the cloud platform. So there is no way to ensure that the task is deployed to the host with the maximum amount of resources each time. Suppose that the task asked by the user is arranged to a randomly selected host each time. If the amount of resources required by a task is larger than the amount of resources available to the host, the host will not be able to efficiently execute the task. This will cause the task assignment event to fail. If the amount of resources asked by a task is very close to the amount of resources available on the physical host on which it is executed, the time spent processing the task will be longer. When a new cloud platform continuously collects task, it will cause the cloud platform's result to be unbalanced and the calculation results cannot be returned to the user in a timely and efficient manner.

To realize load balancing of cloud computing platform in big data processing, the method of finding the optimal load balancing physical host in the algorithm cycle is adopted at present. The purpose of the system design is to seek the best load-balancing host for the current deployment problem within one algorithm cycle [7]. This optimal load balancing strategy, which is overly focused on the current deployment problem, has certain limitations, it will make the system less efficient and the users waiting time unnecessarily prolonged. Load balancing is considered an effective means, but it is not the ultimate goal in itself. Although the resource requests of tasks accepted by the cloud service platform are real-time and strict, the available remaining numbers of computing resources in the service center are always much larger than the resource numbers currently required. Therefore, the instant service performance of the task is easily satisfied. It is almost impossible for the total resource of all task demands collected in one processing cycle to close the total available resource numbers in the cloud computing platform. It is only necessary that the load balance of the whole system is optimal during the task processing. There is no need to try to ensure optimal load balancing from start to finish in real time after every algorithm cycle.

Intelligence decisions over big data are achieved using artificial intelligence paradigms [8]. Both input and computation are necessary as artificial intelligence progresses; therefore, the underlying big data and real-time data necessity are known to increase the accuracy of the outcomes obtained. Based on the above ideas, this study aims to implement a heuristic method called TABAI based on artificial intelligence and big data processing. The TABAI method, based on the artificial intelligence algorithm, will obtain an optimal set of physical hosts for each execution of the currently requested task. The TABAI method achieves the best performance and achieves the best utilization of the current cloud platform resources. The TABAI method achieves a global load balancing effect in the time dimension through multiple executions of the TABAI method for a long time. The TABAI method achieves the best efficiency and external service capability of the cloud platform with a small algorithm overhead. The TABAI method determines a restricted value based on the amount of requested resources for the task. The TABAI method calculates the posterior probability of task assignment for the physical host in the cloud computing platform where the restricted value of the available resources is more than the resource-restricted of task. The TABAI method, based on an artificial intelligence algorithm, filters out the class cluster where the host with the maximum task assignment possibility is located. The assignments will be arranged for the hosts in the collection. The TABAI task assignment approach can not only achieve load balancing in the cloud computing platform but also provide users with high-performance external services.

## 2. Related Theoretical Overview and Research

The goal of load balancing is to make sure that the condition of each host in the cloud computing resource pool reaches a balanced state, achieves high availability of the overall cloud computing platform, and avoids bottleneck nodes in the computing system. It can ensure optimal external service performance and efficiency while making full use of cloud computing resources.

Traditional load balancing solutions include static load balancing (SLB) and dynamic load balancing (DLB) [9]. The SLB algorithm has the disadvantage that it cannot reflect the dynamic load changes of the host cluster. Most of the existing open source IaaS platforms are based on static load balancing methods for resource allocation. For example, the Eucalyptus platform uses the SLB algorithm to achieve load balancing [10]. Wei et al. [11] adopted the SLB algorithm to determine the host weights and used physical hosts with the smallest weight ratio to deploy virtual machines. The effect of static scheduling strategy is not ideal in large-scale cloud computing platforms with strong resource heterogeneity and large differences in user requirements. The design idea of the TABAI method proposed in this study stems from reducing unnecessary computational complexity. The TABAI method is an efficient method capable of dynamic load balancing.

The DLB is a traditional NP entirely combinatorial optimization issue [12]. Dynamic load balancing usually uses heuristic dynamic algorithms to achieve efficient load balancing in real-time by scheduling resources and workloads. The Load Receiver Strategy (LRS) algorithm uses a greedy strategy: light loads are collected and scheduled first [13]. Xu et al. [14] designed a new combinatorial model to dynamically optimize the objective. Lau et al. [15] designed a heuristic load assignment method based on the integration of heavy and light loads to achieve dynamic load balancing with less communication overhead. Dynamic load balancing algorithms usually cannot satisfy both the performance of greedy selection and the properties of optimal substructure. The dynamic load balancing algorithm often obtains the local optimal solution, and the algorithm complexity is high, and the effect of solving the load distribution problem in the big data cloud platform is not ideal.

The load balancing strategy used by the VMware virtualization platform is Distributed Resource Scheduling (DRS) [16]. When VMware uses the DRS method to select physical hosts, it selects a placement strategy that improves overall load balancing by judging the load situation of each host [17]. DRS monitors the load status of all hosts in real time and uses VMware VMotion technology to dynamically schedule virtual machines. DRS realize dynamic optimization of load balancing through dynamic migration of virtual machines. Piao and Yan [18] designed a network aware virtual machine assignment method based on the principle of minimizing data transmission time and improved the performance of the cloud computing platform. However, this method may result in a lower application of host resources while increasing the operational overhead of the cloud computing platform. Sonneck et al. [19] designed a virtual machine dynamic scheduling strategy to minimize communication overhead and achieve load balancing. This strategy dynamically adjusts virtual machine placement by monitoring network affinity.

Shrivastava et al. [20] propose a method to deploy virtual machines that takes on strong application dependencies. This method aims to optimize the management effect of the cloud computing platform and improve its performance and efficiency through virtual machine management. However, this method does not consider load balancing and overhead issues. Rahman and Graham [21] designed a hybrid algorithm combining dynamic and static resource pre-allocation scheduling to increase the computing power of cloud computing platforms. The algorithm adjusts the static initial placement of virtual machines by using dynamic migration in response to changing load environments. Dupont et al. [22] designed a virtual machine rescheduling framework for overall load balancing in cloud computing. The framework achieves load balancing optimization by calculating the optimal placement of virtual machines. Zhao et al. [23] designed a method called MOGA-LS to achieve dynamic load balancing.

Based on the above literature analysis, it is found that the existing work focuses on how to achieve real-time load balancing within an algorithm cycle, that is, the starting point of system design is to find the optimal load balancing physical host for the current deployment problem within an algorithm cycle. This optimal load balancing strategy, which is overly focused on the current deployment problem, has certain limitations, it will make the system less efficient and the user's waiting time unnecessarily prolonged. Load balancing is considered to be an effective means to provide users with satisfactory service performance while maximizing the availability and utilization of the entire cloud system, but it is not the ultimate goal in itself. Since the total amount of resources requested by all tasks collected in one processing cycle is almost impossible to approach the current total available resources in the cloud computing platform, it is sufficient as long as the entire system tends to a long-term optimal load balance. Based on the above ideas, this paper implements a task deployment method for long-term load balancing of cloud platforms based on artificial intelligence from the perspective of the long-term operation of cloud platforms. This method achieves a global load balancing effect in the time dimension through multiple executions for a long time and then achieves the best efficiency and external service capability of the cloud platform with a small algorithm overhead.

## 3. Proposed Methodology

### 3.1. Asking Questions

In the IaaS cloud processing platform, when a user submits a task, the system will call the task assignment module to deploy the corresponding task on the physical host of the cloud resource pool. In most applied cloud platforms, the system usually randomly selects a physical host with sufficient available resources to deploy the task. However, when a task is deployed to a certain host and the amount of resources required by the task is almost the same as the amount of resources currently available on the physical host, the service effect and computing power of the physical host are reduced due to the increased workload of the physical host. At the same time, it will not only lead to the unbalanced load of the cloud processing platform, but also decrease the external service performance and efficiency of the cloud system. Obviously, different task assignment methods under different cloud platforms will achieve different system load distributions, which will make the computing efficiency and external service capabilities of cloud platforms vary greatly. There is no doubt that a cloud computing platform for the efficient processing of big data needs to be equipped with the best task assignment strategy to achieve the best task execution and service effect. This strategy enables the cloud platform to have better load balancing capabilities and optimize cloud computing efficiency.

### 3.2. Analysis and Design Process

**Figure 1:**
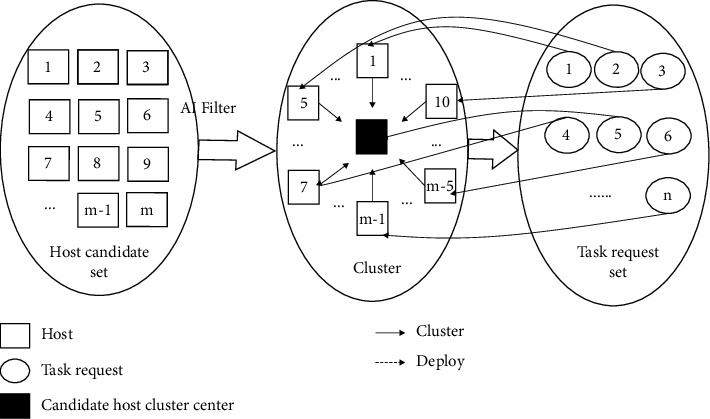
The strategy of task assignment is based on artificial intelligence.

The TABAI method proposed in this study is a heuristic task arrangement method for big data cloud platforms based on artificial intelligence. Its main idea is to combine Bayesian theory with clustering ideas. This method realizes high-performance and high-efficiency load balancing on cloud computing platforms.  Figure 1 shows the task assignment strategy based on artificial intelligence.

First, those physical hosts whose remaining resources are larger than the maximum resource requirements requested by all tasks are filtered out to form a choice set that satisfies the properties constraints. Then, the *k* hosts in the above alternative set are considered as *k* objects to be clustered. Assign a priori probability to the hosts in the set, and calculate the maximum posterior probability of every host using Bayesian theory. The posterior probability of every host executing tasks, the remaining CPU and the amount of memory resources are taken as the three attribute values. TABAI uses these three attribute values to calculate the similarity between all other physical hosts and this host with maximum posterior probability. A threshold is determined based on these similarity values. The host classification is performed according to the threshold value to determine the candidate physical host set. Finally, TABAI deploys the tasks requested by the user to these physical hosts for execution.

### 3.3. Implementation of TABAI

The implementation of the TABAI method is described in detail below.

**Step 1 .**

There are a large number of hosts in the IaaS cloud processing platform. This study assumes that there are *m* hosts in the system, and assigns a performance-restricted value to measure the remaining available computing capacity of each host. The best clustering effect is achieved by minimizing the candidate set and the circumstances that the chosen host does not satisfy the resources required by the task request is prevented. The calculation formula of the performance restricted value *L*_*i*_ of each physical host *i* is as follows

(1) $$\begin{array}{c} L_{i}=\alpha L_{c}^{i}+\beta L_{mem}^{i}\text{,} \end{array}$$

(2) $$\begin{array}{c} \alpha +\beta =1\text{.} \end{array}$$

In equation (1), *α* represents the CPU weight value; *β* represents the memory weight value; obtained through intelligent learning of BP neural network. *L*_mem_^*i*^, *L*_*c*_^*i*^ and *L*_*i*_ are the remaining memory resources, CPU resources, and calculating capacity of the host *i* in turn.

NPH = {} is regarded as an null set, and the property restrain value of the task request set TR is the most request resource in the TR.

(3) $$\begin{array}{c} L_{Mreq}={\max_{i=1}^{n}\;R}_{i}\text{,} \end{array}$$

In equation (3), *L*_*M*req_ is the maximum requested resource. *R*_*i*_ is the demanded resource number of the ith task. Constrain value *L*_*i*_ and performance restricted value *L*_*M*req_ comparison, if *L*_*i*_ greater than *L*_*M*req_, host *I* will be placed in NPH. Finally, a set of candidate hosts NPH = {nph1, nph2,…, nph*m*′}, *m*′ ≤ *m*, is obtained as the following clustering process set of physical host candidates.

**Step 2 .**

Resource-rich hosts are selected and placed in the NPH set according to the resource restricted value requested by the task. However, the host in the NPH set cannot determine whether the CPU resource or the memory resource satisfies the resource constraint value of the task request and is selected. Their *L*_*i*_ values are also large, but in fact do not meet the requirements. In order to fully utilize of the preponderance of the weighted sum of multi-class resources and overcome the shortcomings of some unreasonable hosts, in this study, Bayesian network and cluster analysis algorithms are used to realize host selection and load balancing. The posterior probability of every host being selected in NPH is acquired through a Bayesian probability model. Define event A for these requested tasks to be executed on some physical host. An event *B*_*i*_ is regarded as the host *i* is selected for processing the requested task. The ratio of the maximum requested resources *L*_*M*req_ of the tasks received within one ∆*t* of the cloud computing platform to the remaining computing capacity, *L*_*i*_ of the present host *i* included in the NPH, is taken as a priori probably value. This ratio value is the opposite of the physical meaning intended for this study. From a performance and load balancing perspective, a physical host with more remaining resources should be better suited to handle tasks. This probability value is obtained by.

(4) $$\begin{array}{c} P\left(\left.A\right|B_{i}\right)=1-\frac{L_{Mreq}}{L_{i}}\text{.} \end{array}$$

The probability of selecting a host in the set NPH containing *m*′ hosts is

(5) $$\begin{array}{c} P\left(B_{i}\right)=\frac{1}{m^{\prime }}. \end{array}$$

The posterior probability equation (6) of the host *i* can be obtained through the Bayesian probability model and equations (4) and (5):

(6) $$\begin{array}{c} P\left(\left.B_{i}\right|A\right)=\frac{\left\{\left(L_{i}-L_{Mreq}\right)L_{1}\ldots L_{i-1}L_{i+1}\right\}}{\left\{m^{\prime }L_{1}L_{2}\ldots L_{m^{\prime }}-L_{Mreq}\left(L_{2}L_{3}\ldots L_{m^{\prime }}+\ldots L_{1}L_{2}\ldots L_{i-1}L_{i+1}\ldots L_{m^{\prime }}+\right)\ldots L_{1}L_{2}\ldots L_{m^{\prime -1}}\right\}}\text{.} \end{array}$$

**Step 3 .**

Three attributes of the host *i* are obtained through the above calculation, including the posterior probability, *P*_*i*_, the amount of remaining CPU resources *L*_*c*_^*i*^ and the amount of remaining memory resources, *L*_mem_^*i*^. Select the host nph_*j*_ with the maximum a posteriori probability in the NPH as the cluster center. The similitude between any host and the cluster center is obtained by

(7) $$\begin{array}{c} S=\frac{1}{\sqrt{{\left(P_{i}-P_{j}\right)}^{2}+{\left(L_{c}^{i}-L_{c}^{j}\right)}^{2}+{\left(L_{mem}^{i}-L_{mem}^{j}\right)}^{2}}}\text{.} \end{array}$$

If *P*_*i*_=*P*_*j*_, *L*_*c*_^*i*^=*L*_*c*_^*j*^, and *L*_mem_^*i*^=*L*_mem_^*j*^, then *S* is given a great similarity value. *P*_*j*_ represents the posterior probability of host nph_*j*_. *L*_*c*_^*j*^ is its second attribute value, the amount of remaining CPU resources. *L*_mem_^*j*^ is its third attribute value, the amount of remaining memory resources.

**Step 4 .**

The recognition value between nph_*j*_ and other objects in NPH is obtained by equation (7). The threshold *U*_U_^S^ is assigned judging by the recognition value. If the recognition value *S* is greater than *U*_U_^S^ , add it to NPH′ = {}. By analogy, the final candidate set clustering result NPH′ is constructed, that is, NPH′ = {nph1′, nph2′,…, mph′} (*q* ≤ *m*′ ≤ *m*).

**Step 5 .**

Based on the principle of FIFO task assignment, the host with the largest *L*_*i*_ value in the current NPH′ set is deployed to the tasks in the TR. The time interval from when the TABAI algorithm is invoked to generate the final arranged solution is taken as the time period Δ*t* to be dispatched for the next task. The algorithm takes the number of tasks processed in the last time interval Δ*t* as the workload for the next round of processing.

**Step 6 .**

Cycle the above steps.

### 3.4. Discussion on TABAI

The reason why the procedure of obtaining the priori probably value is consistent with that applied by Bayes' theorem is that Bayes' theorem provides an efficient method of revising the primeval estimate by utilizing the gathered information. Before selecting a host, subjects have a judgment about each hypothesis, which is called a priori probably value. The selected host may not necessarily be able to perform the requested task.

Through the analysis of the artificial intelligence algorithm, the TABAI method can find the optimal host set for each task assignment. Provide efficient long-term load balancing services for big data processing requested by users.

## 4. Analysis and Discussion

The following verify and analyze the TABAI method, the research compares it with the DLB assignment method, the DRS assignment method and the random assignment method, (RD) in the following experiments: (1) MakeSpan; (2) Standard deviation to gauge the effect of load balancing; (3) measure the handling capacity of outbound service quality; (4) the error rate of task arrange events; (5) the percentage increase in the standard deviation value.

The research uses the CloudSim [24] simulator as the test platform, which can realize the cloud environment of this research by providing the real-time addition and deletion of data center entities. On the CloudSim platform, this study compares TABAI, RD, DRS, and DLB. The emulation results show that the TABAI not only has a smaller number of task assignment failures, but also achieves a better load balancing effect on the cloud computing platform, especially when deploying large scale continuous task requests.

### 4.1. Experimental Scene

To obtain better emulation functions and obtain satisfactory experimental results, this study uses CloudSim to simulate a cloud computing platform whose resource pool consists of 100 physical hosts equipped with different available computing resources. Each batch of 24 batches of task requests, consisting of 50 tasks with different resource requirements, is simulated to arrive at the cloud computing platform continuously. The TABAI module will be triggered and called to periodically capture the resource status information of the cloud resource pool. Setting the initial Δt contains 5 task requests.

### 4.2. Comparison of MakeSpan

**Figure 2:**
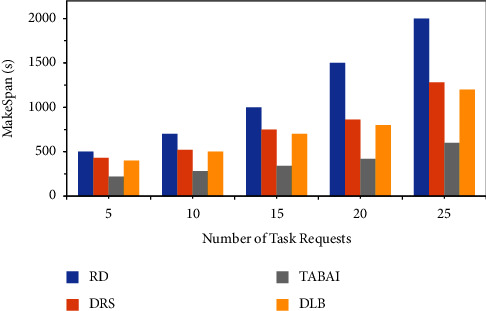
Contrast of MakeSpan between RD, DLB, DRS, and TABAI.

In this set of experiments, the time required for TABAI to perform MakeSpan processing task sets RD, DRS, and DLB will be compared.  Figure 2 shows the experimental results of the three methods. The MakeSpan value will increase because the number of requested tasks will necessarily take more time to process and execute. RD essentially randomly deploys the asked tasks to the hosts of the cloud processing platform. The increase in the number of task requests of the RD method will cause the execution performance of the system to decrease faster, and the task execution time will also increase relatively faster. The DRS method will select a placement strategy that can improve the overall load balance by judging the load status of each physical host. The DRS task execution time will also grow relatively faster. DLB methods only infer upcoming task requirements based on historical records and knowledge bases. DLB decides the task assignment scheme by calculating the benefit numerical of system load balancing. It is found that the increase in tasks will lead to a decrease in task processing performance and a rise in time overhead. However, it is smaller compared to the RD method. In each iteration, TABAI will choose the optimal set of hosts to assign and process tasks to reduce unnecessary communication overhead and maximize the computing performance of the physical cluster. The time to process a task increases at a pace with the amount of demanded tasks raise. With the same number of asked tasks, TABAI has a smaller processing time than DLB, DRS, and RD. As shown in Figure 2, TABAI achieves a relatively smaller MakeSpan value for task processing under the same conditions. This group of experiments shows that TABAI performs low cost and optimization load balancing on the big data cloud computing platform while ensuring the execution performance and efficiency of tasks.

### 4.3. Contrast of Load Balancing Effects

**Figure 3:**
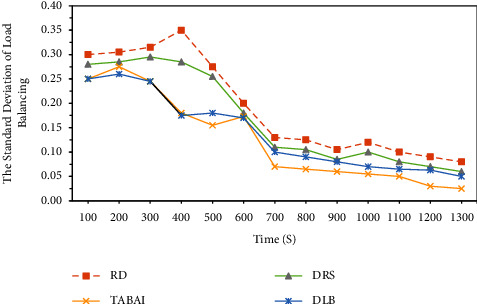
Comparison of load balance between RD, DLB, DRS, and TABAI.

This group of simulations compared the load balancing effects achieved by RD, DLB, DRS, and TABAI on the cloud computing platform over time. The standard deviation value mentioned above for measuring the degree of load balancing is used here to perform the experiment. It can be seen from Figure 3 that the smaller the standard deviation value, the better the load balancing of the cloud computing platform. As shown in Figure 3, the standard deviation of the RD, DRS, method is more than the other two deterministic placement methods over time. For example, when the time is 400 s, the standard deviation of the RD strategy is 0.35, the DRS is 0.28, and the standard deviation of the TABAI and DLB strategies is 0.17. The values of TABAI and DLB gradually decreased. In the beginning, the standard deviation value of DLB was smaller than the values of TABAI. When time = 600 s, the two values are practically the same. However, when time >600 s, the standard deviation value of TABAI always decreased more than that of DLB. The main reason for the analysis is that the TABAI method analyzes the available computing power of the host through artificial intelligence algorithms to ensure that it can achieve the computing resources required by the task. The DLB method first predicts the load of each processor and then deploys tasks according to the knowledge base. It does not handle real-time load status of hosts nor efficiently select the optimal physical host to deploy tasks. This set of simulation results shows that TABAI has a better effect and can effectively increase the resource usage of the cloud computing platform.

### 4.4. Comparison of Exterior Service Capability

**Figure 4:**
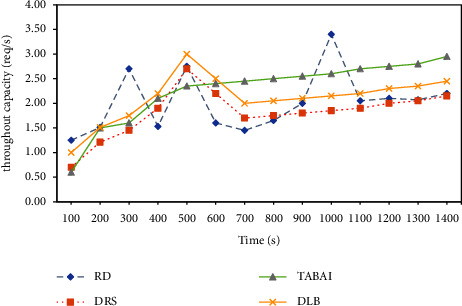
Comparison of external service performance between RD, DLB, DRS, and TABAI.

Since the throughput can delegate the overall merit of the cloud computing platform, it is selected as the estimate criteria to survey the exterior service capability. This set of experiments is in order to measure the performance of the TABAI strategy by comparing the external service performance of the cloud computing platforms configured with these three assignment methods over time. From the simulation results in Figure 4, it can be discovered that the exterior service capability of the three assignment methods is different. When tasks are requested on the cloud computing platform, the compute service capability of RD is outstanding. However, with the increase of time, RD's exterior service capability is unstable and a wave pattern appears. DRS will monitor the load status of all hosts in real time and use VMware VMotion technology to dynamically schedule virtual machines, but the efficiency will decrease significantly with the increase in time. In addition, the exterior service capability of DLB is better than that of TABAI in the initial state. At 700 s, the curve of DLB gradually becomes flat and the throughput smaller. In comparison, the exterior service capability of the TABAI method is longer and more stable. As a result of the host cluster selected by TABAI is composed of some hosts with relatively strong computing power in the current cloud resource pool, so it can provide relatively optimal service performance for the tasks to be deployed. The dynamic load balancing method (DLB) uses historical records and limited predictions to obtain the final assignment plan. The efficiency is difficult to guarantee, and it is prone to secondary assignment events that cause unnecessary extra overhead. From the analysis of the experimental results, it can be seen that the TABAI method shows better usability and efficiency than the existing task assignment methods.

### 4.5. The failed Rate of Task Arrange Events

**Figure 5:**
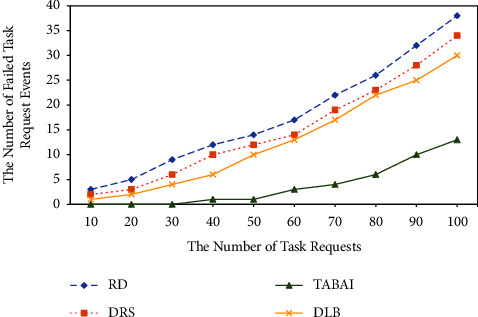
Comparison of the number of failed task arrangement events.

This simulation test simulates dynamic stochastic host failure by using CloudSim to process crash and error events during assignment tasks. The deployment event may fail if the selected target host is in a state of failure or shutdown during the execution of the deployment task and cannot satisfy the task execution request. This set of experiments compares RD, DRS, DLB, and TABAI on the amount of failed deployment tasks.  Figure 5 shows that the number of deployment failures for RD, DRS, and DLB increases as the number of tasks increases. TABAI grows relatively slowly and its mission deployment failures are always lower than RD, DRS, and DLB. The DLB only utilizes the experimental values of the historical knowledge base to achieve the optimal deployment of the current heuristic. DLB does not obtain deployment solutions from a long-term perspective by listening to dynamically available resource information on each candidate host. Therefore, DLB will fail to deploy events when the resource demand of the task is greater than the useable resources of its destination host. Instead, TABAI deploys missions from a long-term perspective. Each time it deploys a task, TABAI ensures that the amount of available resources on each physical host selected is greater than the maximum resource requirement in the task. Therefore, the TABAI method can dynamically and adaptively find the appropriate physical hosts for most task requests in the resource pool, and use the optimal physical cluster to avoid possible host failure events while realizing the long-term load balance optimization of the cloud platform. The overall performance and efficiency of the task are guaranteed.

### 4.6. Contrast of Thorough Load Balancing in Cloud Computing Platform with Different Number of Task Arrange

**Figure 6:**
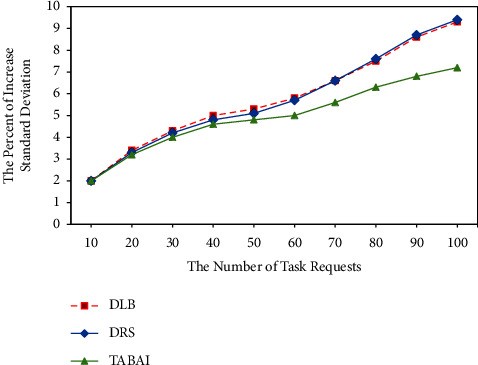
Contrast of standard deviations that increase with the number of task arrange in cloud computing platforms.

As the number of task requests increases, this set of experiments was analyzed by comparing the incremental percentages of DLB, DRS, and TABAI load balancing standard deviation values. As shown in Figure 6, it can be concluded that the cloud computing platform of TABAI for large-scale data processing computing tasks has a better load balancing effect. In addition, it is worth noting that the reason why the RD assignment method is not adopted in this set of experiments is that RD does not possess heuristic information and adaptive ability. RD only randomly deploys tasks to the cloud processing platform. Therefore, the significance of RD participation in validation in this simulation test is limited.

## 5. Conclusion

This paper designs a load balancing method for task processing in the cloud platform based on artificial intelligence and big data analysis technology. TABAI adopts heuristic ideas based on Bayesian theory and the cluster analysis process. TABAI narrows the search by comparing performance constraints and utilizes Bayesian theory to acquire the posterior probabilities for all candidacy hosts. Use artificial intelligence algorithms and big data analysis to select the hosts with the most resources to form an alternative set. And realize the long-term load balance of big data processing on a cloud computing platform. The experimental results show that the TABAI method achieves the long-term global load balance majorisation potential of the cloud platform in the time dimension with relatively small algorithm complexity and can quickly and efficiently deploy and execute instant tasks in the cloud computing platform. Compared with existing work, the TABAI method significantly reduces the number of failures of task deployment events and improves the throughput of data processing. The TABAI method can optimize the computing efficiency and external service capabilities of the cloud platform. The TABAI method can effectively promote the efficient processing of big data under cloud computing. In the future, the TABAI method should have the ability to further select a physical host or more clusters to deploy tasks for the final set of physical hosts so as to realize the optimization of the final host cluster, so as to further improve the efficiency of users and cloud computing platforms.

## Data Availability

The data used to support the findings of this study are available from the corresponding author upon request.
